# SARS-CoV-2 Vaccination Coverage and Factors Associated with Low Uptake in a Cohort of People Living with HIV

**DOI:** 10.3390/microorganisms10081666

**Published:** 2022-08-18

**Authors:** Daniel Kwakye Nomah, Josep Maria Llibre, Yesika Díaz, Sergio Moreno, Jordi Aceiton, Andreu Bruguera, Maria Gutiérrez-Macià, Arkaitz Imaz, Paula Suanzes, Gemma Navarro, Amat Orti, Jose Maria Miro, Jordi Casabona, Juliana Reyes-Urueña, the PISCIS Study Group

**Affiliations:** 1 Centre Estudis Epidemiològics Sobre les Infeccions de Transmissió Sexual i Sida de Catalunya (CEEISCAT), Dept Salut, Generalitat de Catalunya, 08916 Badalona, Spain; 2 Departament de Pediatria, d’Obstetrícia i Ginecologia i de Medicina Preventiva i de Salut Publica, Universitat Autònoma de Barcelona, 08193 Barcelona, Spain; 3 Institut d’Investigació Germans Trias i Pujol (IGTP), 08916 Barcelona, Spain; 4 Hospital Universitari Germans Trias i Pujol, 08916 Badalona, Spain; 5 Hospital de la Santa Creu i Sant Pau, 08019 Barcelona, Spain; 6 Bellvitge Biomedical Research Institute (IDIBELL), University of Barcelona, Hospital Universitari de Bellvitge, 08907 L’Hospitalet de Llobregat, Spain; 7 Vall d’Hebron Research Institute (VHIR), Hospital Universitari Vall d’Hebron, 08935 Barcelona, Spain; 8 Unitat de VIH/SIDA, Corporació Sanitària i Universitària Parc Taulí-Universitat Autònoma de Barcelona, 08208 Barcelona, Spain; 9 Hospital de Tortosa, Verge de la Cinta, 43500 Tortosa, Spain; 10 Hospital Clínic-Institut d’Investigacions Biomèdiques August Pi i Sunyer, University of Barcelona, 08036 Barcelona, Spain; 11 CIBER de Enfermedades Infecciosas (CIBERINFEC), 08036 Barcelona, Spain; 12 CIBER Epidemiologia y Salud Pública (CIBERESP), 08036 Barcelona, Spain

**Keywords:** HIV, severe acute respiratory syndrome coronavirus 2 (SARS-CoV-2), coronavirus disease 2019 (COVID-19), vaccination, vaccine effectiveness

## Abstract

People living with HIV (PLWH) are prioritised for SARS-CoV-2 vaccination due to their vulnerability to severe COVID-19. Therefore, the epidemiological surveillance of vaccination coverage and the timely identification of suboptimally vaccinated PLWH is vital. We assessed SARS-CoV-2 vaccination coverage and factors associated with under-vaccination among PLWH in Catalonia, Spain. As of 11.12.2021, 9945/14942 PLWH (66.6%) had received ≥1 dose of a SARS-CoV-2 vaccine. Non-Spanish origin (adjusted odds ratio (aOR) 0.64, 95% CI 0.59–0.70), CD4 count of 200–349 cells/μL (aOR 0.74, 95% CI 0.64–0.86) or 350–499 cells/μL (aOR 0.79, 95% CI 0.70–0.88), detectable plasma HIV-RNA (aOR 0.61 95% CI 0.53–0.70), and previous SARS-CoV-2 diagnosis (aOR 0.58 95% CI 0.51–0.65) were associated with under-vaccination. SARS-CoV-2 diagnosis (437 [9.5%] vs. 323 [3.5%], *p* < 0.001), associated hospitalisations (10 [2.3%] vs. 0 [0%], *p* < 0.001), intensive care unit admissions (6 [1.4%] vs. 0 [0%], *p* < 0.001), and deaths (10 [2.3%] vs. 0 [0%], *p* < 0.001) were higher among unvaccinated PLWH. Vaccination coverage was lower among PLWH with a CD4 count >200 cells/μL, detectable plasma HIV-RNA, previous SARS-CoV-2 diagnosis, and migrants. SARS-CoV-2 diagnosis, associated hospitalisations, and deaths among PLWH were lower among the vaccinated compared with the unvaccinated. SARS-CoV-2 vaccination prioritisation has not completely reached vulnerable PLWH with poorer prognosis. This information can be used to inform public health strategies.

## 1. Introduction

The World Health Organization (WHO) [1] and the US Center for Disease Control and Prevention (CDC) [2] recommend that people living with HIV (PLWH) are prioritised for SARS-CoV-2 vaccination because of the evidence of their vulnerability to severe COVID-19. Older people infected with HIV, and those with comorbidities, detectable HIV viremia, or advanced immunodeficiency are especially susceptible to a poor COVID-19 prognosis [2,3]. SARS-CoV-2 vaccines provide an opportunity to minimise transmission and severe clinical outcomes from COVID-19, proving their efficacy in clinical trials [4,5] and in real-world settings [6,7]. In an earlier WHO report, out of 100 pooled countries, 40 had prioritised PLWH, especially those with a lower CD4 cell count, for vaccination against SARS-CoV-2 [8].

As of the time of writing, the European Medicines Agency has authorised the Pfizer-BioNTech, Moderna, AstraZeneca/Oxford, and Janssen SARS-CoV-2 vaccines for use in the European Union [9].

These authorised vaccines have no contraindications for use among PLWH, and studies among PLWH receiving mRNA- and adenovirus-based vaccines have shown no safety concerns [10,11]. There are conflicting data regarding the response elicited by SARS-CoV-2 vaccines in PLWH. Some studies have found no difference in anti-SARS-CoV-2 IgM, IgG, and IgA antibody kinetics, peak titres, or neutralisation activity in PLWH compared with individuals who are HIV-negative [12]. However, other studies found that humoral and cellular immune response in PLWH correlates with the CD4 cell count levels with response being significantly weaker in those with a CD4 < 200 cells per μL [13].

In Spain, a technical working group was set up to provide strategic recommendations on the mass vaccine rollout with the objective of reducing COVID-19 morbidity and mortality and protecting the most vulnerable groups [14]. The national vaccination program started on 27 December 2020, initially prioritising older adults, health and socio-sanitary professionals, residents and staff of senior and disability care homes, and highly dependent people not living in care homes, due to vaccine shortage [14]. The vaccines were originally administered in hospitals and primary care centres, and subsequently at established mass vaccination centres. Regardless of the place of administration, all vaccination information is recorded in the individuals’ primary care records. By 31 August 2021, the Spanish government’s target of fully vaccinating 70% of the total population had been achieved [15].

The vaccination campaign prioritised the population based on older age, chronic comorbidities, and the nature of jobs, not on HIV seropositivity [14]. On 21 March 2021, PLWH with a CD4 cell count fewer than 200 cells per μL and those less than 60 years old with chronic comorbidities were included in vaccination priority groups [14]. Nevertheless, there is little information on how vaccines reached specific vulnerable sub-groups.

Reports from previous studies show lower rates of vaccination for other infectious diseases among PLWH compared with the general HIV-negative population, despite their increased risk of infections [16]. Some of the concerns raised by PLWH about vaccinations include fear of clinical side effects, uncertainty about worsening the prognosis of HIV infection, and worry about poor immune response due to their compromised immune systems [17,18].

The epidemiological surveillance of vaccination coverage among PLWH and the timely identification of suboptimally-vaccinated sub-groups is important because social and cultural differences present in this population could make vaccines inaccessible to them, according to reports of vaccine hesitancy in this population [19]. To address this in Catalonia, we assessed the SARS-CoV-2 vaccination coverage in the PISCIS cohort of PLWH in Catalonia, Spain, to describe the distribution and identify sociodemographic, clinical, and epidemiological factors associated with low vaccination uptake. Additionally, we compared SARS-CoV-2 diagnosis and associated clinical outcomes among vaccinated and unvaccinated PLWH without a previous SARS-CoV-2 infection in our cohort.

## 2. Materials and Methods

### 2.1. Study Design and Participants

We conducted a retrospective study leveraging the unique dataset of the PISCIS cohort linked with several administrative health databases (primary care, SARS-CoV-2 vaccination, laboratory, emergency room use, infectious diseases surveillance registries, in-patient hospitalisation, pharmacy, and mortality) under the Public Data Analysis for Health Research and Innovation Program of Catalonia, Spain (PADRIS) [20]. Detailed information about the PISCIS cohort and PADRIS has been described in a previous study [3]. Briefly, the PISCIS cohort is an open, prospective, multicentre, population-based, observational cohort study that follows PLWH aged ≥16 years receiving care at 16 collaborating hospitals in Catalonia. The cohort, which was started in 1998, includes about 80% of all PLWH in Catalonia. The study period was between 27 December 2020, the day that the National Vaccination Program started in Spain, and 11 July 2021. For the purposes of this study, we only included patients who were on clinical follow-up (patients who had used the public healthcare system in the last 18 months).

### 2.2. Procedures

We extracted data on vaccination (type and dates of vaccination), comorbid conditions, SARS-CoV-2 diagnosis confirmed by nucleic acid amplification test (NAAT) and/or antigen detection, and associated clinical outcomes (hospital admissions (more than 24 h with suspicion of respiratory infection and any of the following signs: dyspnea, tachypnea, hypoxemia, asphyxia, or hyperventilation) and intensive care unit (ICU) admission (suffered respiratory failure or sepsis) and death) from the PADRIS. The primary outcome was the reception of a SARS-CoV-2 vaccine (as a binary: vaccinated or unvaccinated). In our analysis, participants were considered vaccinated if they had received a first dose of the Pfizer-BioNTech, Moderna, or Oxford-AstraZeneca SARS-CoV-2 vaccines or the single dose of the Janssen SARS-CoV-2 vaccine. Fully vaccinated individuals were those who had received at least two doses of the Pfizer-BioNTech, Moderna, or Oxford-AstraZeneca SARS-CoV-2 vaccines or a single dose of the Janssen SARS-CoV-2 vaccine. We included data of potential confounders to enable adjusted analyses. Data on sociodemographic characteristics (age, sex, country of origin, and socioeconomic deprivation), HIV-associated variables (median years since HIV diagnosis, HIV exposure risk groups (people who inject drugs (PWID), men who have sex with men (MSM), male heterosexual, female homo/hetero/bisexual), median years on antiretroviral therapy (ART), current ART, most recent CD4 cell count (categorised <200 cells per μL, 200–349 cells per μL, 350–499 cells per μL, and ≥500 cells per μL), plasma HIV-RNA (detectable and undetectable (HIV-RNA of ≤50 copies/mL)), chronic comorbidities, and previous laboratory-confirmed SARS-CoV-2 diagnosis were included. We used the international classification of diseases clinical modifications 9 (ICD-9-CM) and 10 (ICD-10-CM) to extract chronic comorbidities. The ICD classification system was modified to group the most prevalent chronic comorbidities in our population into 11 groups (Supplementary Tables). We counted the number of comorbidities and categorised them into no comorbidities, one comorbidity, two comorbidities, three comorbidities, and four or more comorbidities. We classified socioeconomic deprivation in tertiles (least deprived, mildly deprived, and most deprived) according to the socioeconomic deprivation level index of the Catalan government based on health area of residence (ABS) [21].

### 2.3. Statistical Analysis

We described the baseline characteristics of the vaccinated and unvaccinated groups using proportions. We presented descriptive statistics as the median and interquartile ranges (IQR) for continuous variables and frequencies for categorical variables. Proportions for categorical variables were compared using the χ2 or Fisher’s exact test where appropriate. Continuous variables were compared using the Mann–Whitney U test. We used univariable and multivariable logistic regression models to assess the factors associated with vaccination coverage. In the multivariable model, we adjusted for sex, age, country of origin, socioeconomic deprivation, HIV-exposure group, CD4 levels, plasma HIV RNA, number of chronic comorbidities, and previous SARS-CoV-2 diagnosis. We calculated odds ratios (OR) with 95% confidence intervals (CI) to assess the strength of association. We compared SARS-CoV-2 diagnosis among vaccinated and unvaccinated PLWH without a previous SARS-CoV-2 diagnosis. Among those with confirmed SARS-CoV-2 diagnosis, we compared associated hospital admissions, ICU admissions, and death between the two groups. Records of missing values for adjustment covariates were excluded in the adjusted analyses, as there were few of them and they were not expected to affect estimates significantly. The level of significance of *p* was set at <0.05. We used the R studio software version 4.0.2 to perform all the analyses.

### 2.4. Ethics Statement

The Institutional Review Board of Germans Trias i Pujol Hospital, Badalona, Spain has approved the PISCIS cohort study (EO-11-108). Patient-level information obtained from PADRIS was anonymised and deidentified before the analysis. This study follows the Strengthening the Reporting of Observational Studies in Epidemiology (STROBE) guidelines [22].

## 3. Results

### 3.1. Characteristics of the Study Population

A total of 14,942 PLWH were on clinical follow-up in our cohort as of December 2020. These consisted of 12,257 (82.0%) males, with a median age of 46.4 years. The major transmission group was MSM (52.7%), followed by heterosexual males (13.8%), and 6132 (41.0%) were of non-Spanish origin. Almost half (48.1%) of our study population had a high socioeconomic class, with minimal socioeconomic deprivation. Those in our study population had lived with HIV for a median duration of 11.0 years (IQR 5.7–17.7 years). The median CD4 count was 680.0 cells/μL, with 3069 (20.5%) having CD4 levels of <200 cells/μL. Plasma HIV-RNA was undetectable in 11,891 (79.6%) patients. Regarding comorbidities, 4849 (32.5%) PLWH were without comorbidities (Table 1).

### 3.2. SARS-CoV-2 Vaccination

**Figure 1:**
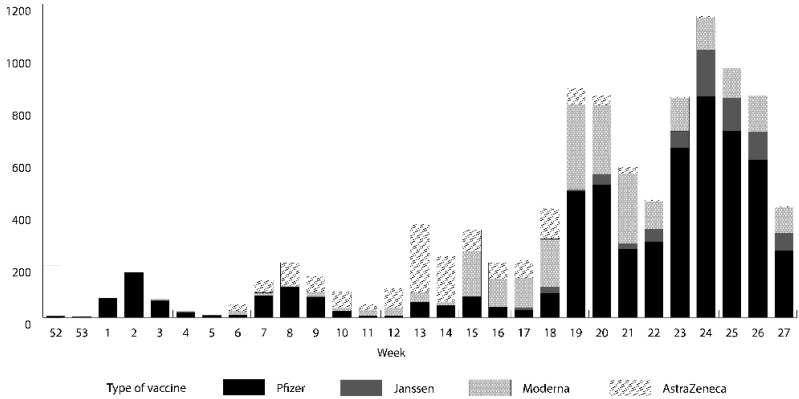
Number of vaccinated people living with HIV in Catalonia by manufacturer and calendar week (Week 52: 21–27 December 2020; Week 27: 5–11 July 2021).

Between 27 December 2020 and 11 July 2021, 9945 out of 14,942 PLWH (66.6%) in our cohort received at least one dose of a SARS-CoV-2 vaccine. The findings showed that 6949 (69.9%) were fully vaccinated and 2996 (30.1%) had received a first dose (Supplementary Tables). Vaccination among PLWH in Catalonia peaked between 14 and 20 June 2021 (week 24) (Figure 1). A majority of our cohort received the Pfizer-BioNTech vaccine (56.7%), followed by Moderna (23.2%), AstraZeneca (12.9%), and Janssen (7.2%).

**Figure 2:**
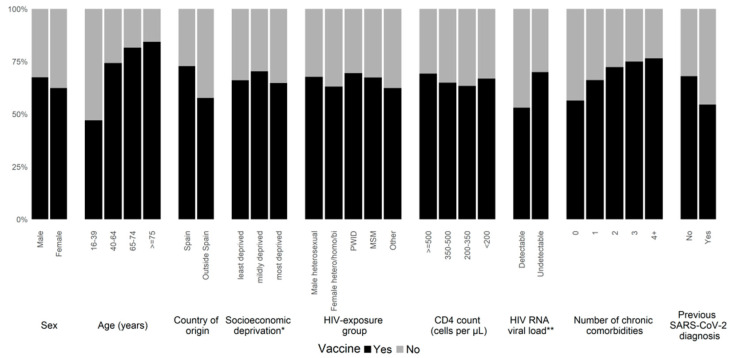
SARS-CoV-2 vaccination uptake according to various sociodemographic and clinical groups in the PISCIS cohort of people living with HIV (27 December 2020–11 July 2021). Abbreviations: SARS-CoV-2, severe acute respiratory syndrome coronavirus 2; PWID, people who inject drugs; MSM, men who have sex with men; * According to the socioeconomic deprivation level index of the Catalan government based on the health area of residence. ** plasma HIV-RNA undetectable: ≤50 copies/mL.

Vaccination was less common among females (62.3%) than males (67.5%) (*p* < 0.001) (Figure 2).

The median age in the vaccinated population was higher (49.0 years, IQR 41.4–55.8) than in the unvaccinated population (40.8 years, IQR 32.8–49.3) *p* < 0.001). The proportion of migrants was higher in the unvaccinated group compared with the vaccinated group (52.0% vs. 35.6%, *p* < 0.001). Vaccination uptake was similar across all socioeconomic deprivation categories (*p* = 0.52). Regarding HIV exposure groups, we observed a slightly higher vaccination uptake among PWID (69.5%), followed by heterosexual males (67.7%), MSM (67.4%), and female hetero/homo/bisexuals (63.0%). CD4 cell levels were higher among the vaccinated than the unvaccinated (median 689.0 cells per μL vs. 662.0 cells per μL, *p* < 0.001). Vaccination coverage was higher among PLWH with undetectable plasma HIV-RNA compared with those with unsuppressed HIV viraemia (69.9% vs 53.3%, *p* < 0.001). We found a higher coverage among PLWH with comorbidities than those without (72.5% vs. 57.7%, *p* < 0.001). Vaccination coverage increased with an increasing number of comorbidities (no comorbidities: 56.4%; one comorbidity: 66.2%; two comorbidities: 72.3%; three comorbidities: 75.0%; four or more comorbidities: 76.5%) (Figure 2). Among vaccinated PLWH in our cohort, 1057 (10.6%) had previous SARS-CoV-2 infection, and this was higher among unvaccinated patients (732/4997, 14.7%, *p* < 0.001) (Table 1).

### 3.3. Factors Associated with Low Uptake of SARS-CoV-2 Vaccines

In order to identify sub-groups that could be undervaccinated, we used multivariable logistic regression analysis. After controlling for possible confounding factors, PLWH were found to be less likely to receive the SARS-CoV-2 vaccine if they were born outside of Spain (aOR 0.64, 95% CI 0.59–0.7), had a CD4 cell count of 200–349 cells per μL (aOR 0.74, 95% CI 0.64–0.86) or 350–499 cells per μL (aOR 0.79, 95% CI 0.70–0.88), detectable plasma HIV-RNA (aOR 0.61, 95% CI 0.53–0.70) or a previous SARS-CoV-2 diagnosis (aOR 0.58, 95% CI 0.51–0.65). Vaccine coverage was likely to increase with increasing age groups (40–64 years (aOR 3.01, 95% CI 2.75–3.30), 65–74 years (aOR 3.77, 95% CI 3.01–4.77), and ≥75 years (aOR 5.77, 95% CI 3.27–8.24)), and was associated with male sex (aOR 1.39, 95% CI 1.12–1.72). We also observed a higher odds of vaccine coverage among PLWH with mild socioeconomic deprivation (aOR 1.21, 95% CI 1.08–1.35), MSM (aOR 1.43, 95% CI 1.26–1.62) and those with comorbid conditions (one comorbidity (aOR 1.28, 95% CI 1.16–1.43), two comorbidities (aOR 1.58, 95% CI 1.39–1.78), three comorbidities (aOR 1.58, 95% CI 1.36–1.84), or four or more comorbidities (aOR 1.58, 95% CI 1.37–1.83)) (Table 2).

### 3.4. SARS-CoV-2 Outcomes among Vaccinated and Unvaccinated People Living with HIV

SARS-CoV-2 diagnosis (437 (9.5%) vs. 323 (3.5%), *p* < 0.001), associated hospitalisations (10 (2.3%) vs. 0 (0%), *p* < 0.001), intensive care unit admissions (6 (1.4%) vs. 0 (0%), *p* < 0.001), and deaths (10 (2.3%) vs. 0 (0%), *p* < 0.001) were higher among the unvaccinated PLWH compared with the vaccinated (Table 3).

## 4. Discussion

While our study in this large cohort of PLWH revealed that a majority (66.6%) of the population had received a SARS-CoV-2 vaccine as of 11 July 2021, coverage in some specific groups might not be optimal. We observed lower uptake among those with a CD4 cell count of >200 cells per μL, detectable plasma HIV-RNA, previous SARS-CoV-2 diagnosis, and migrants. Furthermore, SARS-CoV-2 diagnoses, associated hospitalisations, ICU admissions, and deaths were lower among vaccinated PLWH compared with the unvaccinated.

Astounding evidence establishes vaccines as one of the most successful and cost-effective health interventions ever [23]. In the case of COVID-19, the vaccines offer promising prospects to beat a pandemic that has threatened overall stability globally and caused overwhelming morbidity and mortality [24,25,26,27].

The government of Spain set out a vaccination strategy that sought to vaccinate those most vulnerable to SARS-CoV-2 first and met its goal of fully vaccinating at least 70% of the total population by 31 August 2021 [28]. Even though the vaccine rollout started slowly in the first few months in Spain due to a severe vaccine shortage, the procurement of more vaccines by the government, the introduction of mass vaccination centres, and the resolution of logistical barriers accelerated the vaccine uptake steadily from April/May 2021, with the vaccination peak in Spain coinciding with the observed vaccination peak in our cohort. The peak period also coincides with the vaccination period for people aged 40–59 years in Catalonia and Spain [28]. This age group contributes to more than half of the overall PISCIS cohort and that could be an additional reason explaining the peak in vaccinations in June among our population. The 66.6% vaccination coverage observed in our cohort as of 11 July 2021 is similar to that in the Catalonian general population, with a coverage of 67.7% (at least one dose) as of the same week [29].

Regardless of the fairly comparable coverage between the general population and PLWH in Catalonia, almost all persons aged 60 years and above in Spain had received at least one vaccine dose against SARS-CoV-2 as of 14 July 2021 (98.1%) [29], but that was not the case in our cohort (overall 80%). PLWH aged 60 years and above were called from their respective primary care centres and subsequently from the HIV units in the various hospitals, suggesting that the suboptimal coverage in this group is because of hesitancy.

Existing studies have identified migrants as a vulnerable group for COVID-19 in the general population [30] and among PLWH [3]. Not unexpectedly, we found a lower vaccination uptake in this group. Migrants face systemic healthcare access barriers and are affected by varying social determinants of health, which could hamper their access to SARS-CoV-2 vaccines. Some studies have also found higher vaccine hesitancy and low vaccination coverage among migrants and other ethnic minority groups [31]. This finding is concerning and calls for action and further investigation to understand the low acceptance and barriers to healthcare and vaccine access among migrants living with HIV.

We anticipated that all PLWH with CD4 levels <200 cells per μL would be vaccinated by 11 July 2021, because this group is prioritised for vaccination in Spain due to their vulnerability to COVID-19 [14]. In our cohort, however, a third of the population had not received the vaccine as of this date despite being prioritised in public health strategies. We were also concerned to find that unsuppressed HIV viraemia was associated with lower vaccination coverage. Non-HIV suppression and lower CD4 cell counts are associated with drug abuse, social determinants, poor treatment, and follow-up compliance, and hence poor health care in general, and could explain why coverage was lower in these groups. A study from Catalonia identified detectable plasma HIV RNA as a risk factor for poor COVID-19 prognosis [3]. Given the increased risk for COVID-19-associated morbidity and mortality in these clinical groups, addressing SARS-CoV-2 vaccination barriers in these subpopulations is crucial.

Our study revealed that PLWH with a previous SARS-CoV-2 diagnosis were less likely to be vaccinated. As of 11 July 2021, the vaccination protocol for persons less than 65 years of age previously infected with SARS-CoV-2 in Spain was a single dose six months after the confirmed SARS-CoV-2 diagnosis [14]. This could explain why the vaccine coverage is lower among previously infected people.

The lower rates of SARS-CoV-2 diagnosis, associated hospital admissions, ICU admissions, and deaths among vaccinated PLWH compared with the unvaccinated is an important indication of the benefits achieved with SARS-CoV-2 vaccines in this population. Further studies assessing vaccine effectiveness among PLWH and the effect of vaccination on poor clinical outcomes in real-world settings are vital.

Our multicentre cohort study had some limitations. Given the importance of clinical variables in understanding the subgroups with suboptimal vaccination coverage, we excluded patients who had not used the public healthcare system in Catalonia in the past 18 months. We therefore could be over-estimating the vaccination coverage in our population. We also do not have information on individuals who were vaccinated outside the Catalonia autonomous region because, in Spain, the 17 Autonomous Communities are responsible for offering integrated healthcare services to the regional population and managing their public healthcare system. Another important limitation of our study was our inability to evaluate the immunological response to vaccines among PLWH over time and in specific clinical groups such as those with CD4 cells less than 200 cells/mm^3^ and unsuppressed HIV viremia. Vaccination reception can be influenced by level of education, income, and religious beliefs. We were limited by our inability to control for these factors due to the nature of cohort studies among human populations.

In conclusion, our study reveals that a majority (66.6%) of PLWH in our cohort had received at least one dose of a SARS-CoV-2 vaccine as of 11 July 2021 and significantly lower SARS-CoV-2 diagnosis, associated hospitalisations, and deaths were observed among vaccinated PLWH compared with the unvaccinated, mirroring what happened in the general population. Nevertheless, non-Spanish origin, a CD4 cell count >200 cells per μL, detectable plasma HIV-RNA, and a previous SARS-CoV-2 diagnosis were associated with lower vaccination coverage, suggesting that vaccine strategies towards PLWH have been driven by general criteria and not taking specific vulnerability factors into account. Our data confirm the benefits of the SARS-CoV-2 vaccine also to PLWH and reinforce the need to proactively identify PLWH at risk for under-vaccination and to design targeted public health strategies to improve vaccine uptake in these specific socio-demographic and clinical groups.

## Figures and Tables

**Table 1:** Baseline characteristics of people living with HIV in Catalonia according to SARS-CoV-2 vaccine status (December 2020–July 2021).

	Overall Cohort (n = 14,942)	Vaccinated ^x^ (n = 9945)	Unvaccinated (n = 4997)	*p* Value
Characteristic	n (%)	n (%)	n (%)	
**Sex ^a^**				<0.001
Male	12257 (82.0)	8271 (83.2)	3986 (79.8)	
Female	2684 (18.0)	1673 (16.8)	1011 (20.2)	
Missing	1 (0.01)	1 (0.01)	0 (0)	
**Age, median (IQR), y ^b^**	46.4 (38.3–54.2)	49.0 (41.4–55.8)	40.8 (32.8–49.3)	<0.001
**Age category, y ^b^**				<0.001
16–39	4479 (30.0)	2106 (21.2)	2373 (47.5)	
40–64	9593 (64.2)	7124 (71.6)	2469 (49.4)	
65–74	678 (4.5)	553 (5.6)	125 (2.5)	
≥75	192 (1.3)	162 (1.6)	30 (0.6)	
**Country of origin ^c^**				<0.001
Spain	8808 (59.0)	6409 (64.4)	2399 (48.0)	
Outside Spain	6132 (41.0)	3535 (35.6)	2597 (52.0)	
Unknown	2 (0.01)	1 (0.01)	1 (0.02)	
**Socioeconomic deprivation**				
Least deprived	7188 (48.1)	4749 (47.8)	2439 (48.8)	0.52
Mildly deprived	2839 (19.0)	1997 (20.1)	842 (16.9)	
Most deprived	4574 (30.6)	2962 (29.8)	1612 (32.3)	
Missing	341 (2.3)	237 (2.4)	104 (2.1)	
**HIV transmission route**				<0.001
PWID	1727 (11.6)	1200 (12.1)	527 (10.6)	
MSM	7835 (52.4)	5281 (53.1)	2554 (51.1)	
Male heterosexual	2055 (13.8)	1391 (14.0)	664 (13.3)	
Female hetero/homo/bisexual	2008 (13.4)	1266 (12.7)	742 (14.9)	
Other	856 (5.7)	534 (5.4)	322 (6.4)	
Missing	461 (3.1)	273 (2.8)	188 (3.8)	
**CD4 count (cells/μL) category**				<0.001
<200	3069 (20.5)	2051 (20.6)	1018 (20.4)	
200–349	1266 (8.5)	802 (8.1)	464 (9.3)	
350–499	2066 (13.8)	1341 (13.5)	725 (14.5)	
≥500	7833 (52.4)	5425 (54.6)	2408 (48.2)	
Missing	708 (4.7)	326 (3.3)	382 (7.6)	
**HIV-RNA**				<0.001
Detectable	1476 (9.9)	783 (7.9)	693 (13.9)	
Undetectable	11891 (79.6)	8317 (83.6)	3574 (71.5)	
Missing	1575 (10.5)	845 (8.5)	730 (14.6)	
**Number of comorbidities**				<0.001
0	4849 (32.5)	2735 (27.5)	2114 (42.3)	
1	3661 (24.5)	2422 (24.4)	1239 (24.8)	
2	2596 (17.4)	1878 (18.9)	718 (14.4)	
3	1602 (10.7)	1201 (12.1)	401 (8.0)	
≥4	2234 (15.0)	1709 (17.2)	525 (10.5)	
**Previous SARS-CoV-2 diagnosis**				<0.001
Yes	1610 (10.8)	878 (8.8)	732 (14.7)	
No	13332 (89.2)	9067 (91.2)	4265 (85.4)	

Abbreviations: PLWH, people living with HIV; SARS-CoV-2, severe acute respiratory syndrome coronavirus 2; IQR, interquartile range; PWID, people who inject drugs; MSM, men who have sex with men. a Sex at birth as registered in the health registries of the participating hospitals was used. One participant in the overall cohort had an unknown sex. b Age for all patients was as of December 1, 2020. c Country of origin was as indicated by the Public Data Analysis for Health Research and Innovation Program of Catalonia (PADRIS) recorded as Spanish or Non-Spanish. x ≥1 dose of SARS-CoV-2 vaccine.

**Table 2:** Factors associated with SARS-CoV-2 vaccine coverage among people living with HIV in Catalonia in multivariable logistic regression.

Characteristics		OR	aOR ^a^
**Sex**	Female	1.00 (ref)	1.00 (ref)
	Male	1.25 (1.15–1.37)	1.39 (1.12–1.72)
**Age category, y**	16–39	1.00 (ref)	1.00 (ref)
	40–64	3.25 (3.02–3.50)	3.01 (2.75–3.30)
	65–74	4.98 (4.08–6.13)	3.77 (3.01–4.77)
	≥75	6.08 (4.17–9.19)	5.08 (3.27–8.24)
**Country of origin**	Spain	1.00 (ref)	1.00 (ref)
	Outside Spain	0.51 (0.48–0.55)	0.64 (0.59–0.70)
**Socioeconomic deprivation**	Least deprived	1.00 (ref)	1.00 (ref)
	Mildly deprived	1.22 (1.11–1.34)	1.21 (1.08–1.35)
	Most deprived	0.94 (0.87–1.02)	0.97 (0.88–1.07)
**HIV transmission route**	Male heterosexual	1.00 (ref)	1.00 (ref)
	Female hetero/homo/bisexual	0.81 (0.72–0.93)	1.14 (0.88–1.48)
	PWID	1.09 (0.95–1.25)	0.87 (0.74–1.03)
	MSM	0.99 (0.89–1.09)	1.43 (1.26–1.62)
	Other	0.79 (0.67–0.94)	1.06 (0.87–1.30)
**CD4 count (cells/μL) category**	≥500	1.00 (ref)	1.00 (ref)
	350–499	0.82 (0.74–0.91)	0.79 (0.70–0.88)
	200–349	0.77 (0.68–0.87)	0.74 (0.64–0.86)
	<200	0.89 (0.82–0.98)	0.92 (0.83–1.02)
**HIV RNA viral load**	Undetectable	1.00 (ref)	1.00 (ref)
	Detectable	0.49 (0.44–0.54)	0.61 (0.54–0.69)
**Number of comorbidities**	0	1.00 (ref)	1.00 (ref)
	1	1.51 (1.38–1.65)	1.28 (1.16–1.43)
	2	2.02 (1.82–2.24)	1.58 (1.39–1.78)
	3	2.31 (2.04–2.63)	1.58 (1.36–1.84)
	≥4	2.52 (2.25–2.82)	1.58 (1.37–1.83)
**SARS-CoV-2 diagnosis**	No previous SARS-CoV-2 diagnosis	1.00 (ref)	1.00 (ref)
	Previous SARS-CoV-2 diagnosis	0.56 (0.51–0.63)	0.58 (0.51–0.65)

Abbreviations: PLWH, people living with HIV; SARS-CoV-2, severe acute respiratory syndrome coronavirus 2; COVID-19, Coronavirus disease 2019; PWID, people who inject drugs; MSM, men who have sex with men; a Model adjusted for sex, age, country of origin, socioeconomic deprivation, HIV-exposure group, CD4 levels, HIV RNA viral load, chronic comorbidities, and previous SARS-CoV-2 diagnosis.

**Table 3:** SARS-CoV-2 diagnosis and associated clinical outcomes among people living with HIV vaccinated and unvaccinated against COVID-19 (27 December 2020–11 July 2021).

	Total n = 13,662	Unvaccinated PLWH n = 4596	Vaccinated PLWH n = 9066	
	**n (%)**	**n (%)**	**n (%)**	***p***
SARS-CoV-2 diagnosis	616 (4.5)	437 (9.5)	179 (2.0)	<0.001
COVID-19 hospital admissions	10 (1.6)	10 (2.3)	0 (0)	<0.001
COVID-19 ICU admissions	6 (1.0)	6 (1.4)	0 (0)	<0.001
COVID-19 deaths	10 (1.6)	10 (2.3)	0 (0)	<0.001

Abbreviations: PLWH, people living with HIV; SARS-CoV-2, severe acute respiratory syndrome coronavirus 2; COVID-19, Coronavirus disease 2019; ICU, intensive care unit.

## Data Availability

The study protocol is available from Reyes-Urueña (e-mail: jmreyes@iconcologia.net). Statistical code for the analysis can be requested from Yesika Diáz, Sergio Moreno, and Jordi Aceiton (ydiazr@iconcologia.net, smorenof@iconcologia.net, jaceiton@igtp.cat). The data for this study are available at the Centre for Epidemiological Studies of Sexually Transmitted Diseases and HIV/AIDS in Catalonia (CEEISCAT), the coordinating centre of the PISCIS cohort and from each of the collaborating hospitals upon request via https://pisciscohort.org/contacte/.

## Supplementary Materials

The following supporting information can be downloaded at: https://www.mdpi.com/article/10.3390/microorganisms10081666/s1, Table S1: Baseline characteristics of people living with HIV in Catalonia according to reception of a single dose or full vaccination (December 2020–July 2021); Table S2: Eleven groups of the most prevalent chronic comorbidities among people living with HIV in Catalonia with corresponding International Classification of Diseases, Ninth and Tenth Revision, Clinical Modification (ICD-9/10-CM) codes. Table S3: Baseline characteristics of people living with HIV in Catalonia according to SARS-CoV-2 vaccine status (27 December–July 2021).
